# The Effectiveness of Trauma-Focused Psychodrama in the Treatment of PTSD in Inpatient Substance Abuse Treatment

**DOI:** 10.3389/fpsyg.2020.00896

**Published:** 2020-05-19

**Authors:** Scott Giacomucci, Joshua Marquit

**Affiliations:** ^1^Phoenix Center for Experiential Trauma Therapy, West Chester, PA, United States; ^2^Mirmont Treatment Center, Lima, PA, United States; ^3^Bryn Mawr College, Bryn Mawr, PA, United States; ^4^Pennsylvania State University, Brandywine Campus, Media, PA, United States

**Keywords:** psychodrama, trauma, addiction, therapeutic spiral model, relational trauma repair model

## Abstract

This single group pretest-posttest study explores the effectiveness of trauma-focused psychodrama in the treatment of post-traumatic stress disorder (PTSD) at an inpatient addiction treatment center. The results contribute to the limited research bases of both psychodrama and PTSD treatment outcomes in inpatient addiction treatment. The present study supports the potential effectiveness of two trauma-focused psychodrama models, the Therapeutic Spiral Model and the Relational Trauma Repair Model. Findings of the research demonstrate clinically significant reductions in overall PTSD symptoms (over 25% change) and each PTSD symptom cluster (i.e., re-experiencing and intrusion, avoidance and numbing, and hyper-arousal). Additionally, patient satisfaction exit survey data support overall treatment effectiveness and highlight its tolerability, and capacity for establishing emotional safety, connection, and group cohesion. Patients even described the trauma-focused psychodrama treatment approach as enjoyable and helpful. Overall, the results of this study are promising, and support continued clinical application of trauma-focused psychodrama treatment with other subpopulations diagnosed with PTSD. However, the ability to isolate effects of trauma-focused psychodrama in this study is compromised due to the absence of a control group and participants’ involvement in other inpatient treatment services.

## Introduction

Nearly 8% of Americans will meet diagnostic criteria for Post-Traumatic Stress Disorder (PTSD) in their lifetimes, and about 8 million adults during any given year, according to the United States Department of Veteran Affairs (2016). In recent years, the treatment of trauma has become a popular focus in clinical practice and addiction treatment. Research has demonstrated a strong connection between addiction and trauma.

Over 80% of women seeking treatment for Substance Use Disorder (SUD) report a history of physical or sexual assault (Cohen and Hien, 2006). Men with a SUD are 7.2 times more likely to have PTSD than those without a SUD; women with a SUD are 12.4 times more likely to have PTSD than those without a SUD (Creamer et al., 2001). The comorbidity between PTSD and Alcohol Use Disorder (AUD) is even higher with a 36–52% co-occurrence rate (Roberts et al., 2015). The groundbreaking Adverse Childhood Experiences (ACE) study has uncovered a significant connection between childhood trauma and later adult issues with substances, alcohol, mental illness, and other medical issues (Felitti, 2003; Forster et al., 2017). In the addiction treatment industry, many believe that trauma and PTSD cannot be treated concurrently with addiction due to fears of relapse or dropout. This belief is supported by very little empirical evidence (Torchalla et al., 2012; Flanagan et al., 2016); instead, an integrated treatment approach that treats both addiction and trauma has demonstrated the best outcomes (McCauley et al., 2012; Torchalla et al., 2012; Ralevski et al., 2014; Roberts et al., 2015; Morgan, 2019).

Simultaneously, the rapid evolution in neurobiology research has provided clinicians with a more comprehensive understanding of the ways that trauma impacts an individual. As a result, many treatment approaches have been challenged to orient their approaches with these neuroscience findings. Recent research has indicated that when a traumatic memory is activated it appears to significantly impact the functioning of the speech and language centers of the brain (Rauch et al., 1996; van der Kolk, 2014), which theoretically challenges the effectiveness of talk therapy. These neuroscience findings (Siegel, 2012; Cozolino, 2014; van der Kolk, 2014) have been used to suggest that experiential therapy is the treatment of choice when working with PTSD (van der Kolk, 1996, 2014; Hudgins and Drucker, 1998; Hug, 2013; Dayton, 2015; Hudgins, 2017; Giacomucci and Stone, 2018).

## Background

Psychodrama is an experiential approach, often used in psychotherapy, that integrates role-playing techniques, dramatic enactment, and spontaneous improvisation (Moreno, 1946). Classical psychodrama was developed nearly 100 years ago by Dr. Jacob Moreno, who also coined the terms “group therapy” and “group psychotherapy” (Moreno, 1946, 1953, 2019; Nolte, 2014). Moreno’s psychodrama emerged from his early work with children, immigrants, refugees, prostituted women, prisoners, and later severely mentally ill patients at his sanitarium in upstate New York. By 1952, over two dozen Veteran’s Administration Hospitals around the United States had integrated Moreno’s work into their programs (Moreno, 2019). Until 2004, St. Elizabeth’s Hospital outside Washington D.C. was home to a prestigious psychodrama internship program which provided extensive training to psychodramatists from around the world (Buchanan and Swink, 2017). St. Elizabeths Hospital was a major provider of treatment for US military veterans. Although Moreno died in 1974, six years before PTSD was recognized by the American Psychiatric Association in the third edition of the Diagnostic and Statistical Manual of Mental Disorders, his psychodrama was used extensively to treat PTSD in inpatient hospital programs.

In the past few decades, the treatment of PTSD has become a major clinical concern which gave way to two trauma-focused psychodrama models – the Therapeutic Spiral Model (TSM) and Relational Trauma Repair Model (RTR). TSM is a clinically modified version of classical psychodrama which emphasizes safety, containment, and strengths for treating PTSD (Hudgins and Drucker, 1998). TSM is a comprehensive experiential trauma therapy model complete with its own clinical map which is conceptualized in role theory and guided by neurobiology research and attachment theory (Giacomucci, 2018). The TSM model includes six safety structures focused on strength-based experiential sociometry (Giacomucci et al., 2018). RTR is also grounded in the interpersonal neurobiology research and attachment literature offering a variety of experiential group processes ranging from experiential psychoeducation, action-based sociometry tools, and psychodramatic enactments (Dayton, 2015). The RTR model is unique in that it has been adapted for clinical use in shorter groups and offers a potent alternative to full psychodrama sessions for time-limited sessions. RTR offers multiple experiential processes for working with relational trauma that focus on the psychosocial metrics within the group (Giacomucci, 2020a). While a TSM or classical psychodrama would often include multiple roles and scenes, an RTR psychodrama most often only has two or three roles. RTR’s founder, Tian Dayton, describes it as ‘surgical role reconstruction’ which allows trauma survivors to renegotiate internalized trauma scenes for moments of repair (Dayton, 2014).

Experience changes the brain (Siegel, 2012). A psychodramatic experience has the potential of changing the intrapsychic and somatic imprints of trauma by accessing the traumatic neural network and providing the client with a safe way of renegotiating their trauma and facilitating a completion of the central nervous system’s survival responses (fight/flight/freeze) (Levine, 2010; Porges, 2017; Giacomucci and Stone, 2018). Thus, unlike other treatment approaches that focus on symptom control, TSM provides an avenue for renegotiation, integration, and resolution of unresolved trauma or loss (Hudgins, 2000, 2002, 2007, 2017; Giacomucci and Stone, 2018; Giacomucci, 2019, 2020a).

While the current literature on TSM psychodrama is quite limited, preliminary findings suggest possible effectiveness in reducing PTSD symptoms and even resolving trauma (Hudgins et al., 2000; Gow and McVea, 2006; Hudgins and Toscani, 2013; Perry et al., 2016). The currently published research on TSM is limited to a study of individual sessions using the Containing Double, which is a TSM specific intervention, and a multi-day workshop for female service members. There does not appear to be any research published about the use of TSM in inpatient treatment settings. At the same time, the RTR model, does not appear to have any published research demonstrating its efficacy in treatment. This research study will contribute to the growing research base in the psychodrama literature by exploring the effectiveness of trauma-focused (TSM and RTR) psychodrama groups for reducing PTSD symptoms in an inpatient addictions treatment center trauma tract.

Although the research literature on TSM and RTR psychodrama is limited, there have been multiple other studies on psychodrama’s effectiveness published. The most recent systematic review of the psychodrama research states “psychodrama intervention research in the last decade suggests there are promising results” while also highlighting the need for higher quality psychodrama research studies (Orkibi and Feniger-Schaal, 2019, p. 1). Similarly, Wieser, 2007 meta-analysis on the efficacy of psychodrama psychotherapy states that the psychodrama research shows some evidence of positive effects for a variety of mental health disorders, but that more rigorous scientific research needs to be completed. A significant meta-analysis published by Kipper and Ritchie (2003) indicated that psychodrama groups demonstrated an overall effect size “similar to, or better than that commonly reported for group psychotherapy in general” (p. 1). Additionally, they note that “although the initial empirical research on the effectiveness of psychodrama revealed some encouraging results, the data were insufficient” (p. 14). Kellermann’s (1987) synthesis of psychodrama research indicates that while there are limitations to the empirical evidence, “Psychodrama was a very valid alternative to other therapeutic approaches, primarily in promoting behavior change with adjustment, antisocial, and related disorders” (p. 467). Similarly, Rawlinson (2000) concluded that “there is some research evidence to support the use of Psychodrama” and that psychodrama can be used “as a tool for helping people to develop self-esteem, to change elements of their behavior and to develop empathy and social relationships” (p. 93).

Wieser indicates that “neurotic, stress-related and somatoform disorders are the best validated area for psychodrama therapy” (2007, p. 278). Various psychodrama research studies in the addictions field have demonstrated its efficacy in increasing quality of life (Dehnavi et al., 2016) and motivation (Testoni et al., 2018), reducing depression (Dehnavi et al., 2015) and aggression (Nooripour et al., 2016), and for relapse prevention training (Somov, 2008). While the use of psychodrama in the addictions field is increasingly common (Dayton, 2005, 2015; Giacomucci, 2017, 2020a), there have been few studies to date on its effectiveness.

The currently available literature related to psychodrama as a treatment modality supports its efficacy in improving emotional/psychological stability (Schmidt, 1978; Wood et al., 1979; White et al., 1982; Carpenter and Sandberg, 1985; Choi, 2003; Kang and Son, 2004) interpersonal relationships (Petzold, 1979; Shim, 2002; Gow and McVea, 2006; Gow et al., 2011; Bendel, 2017), improving conflict resolution skills (Karatas and Gokcakan, 2009; Karatas, 2011), reducing depression (Hall, 1977; Carbonell and Parteleno-Barehmi, 1999; Costa et al., 2006; Avinger and Jones, 2007; Wieser, 2007; Sharma, 2017; Souilm and Ali, 2017; Erbay et al., 2018), reducing anxiety and panic disorder systems (Hall, 1977; Schramski et al., 1984; Carbonell and Parteleno-Barehmi, 1999; Park and Lim, 2002; Avinger and Jones, 2007; Wieser, 2007; Sharma, 2017; Tarashoeva et al., 2017; Erbay et al., 2018), increased self-esteem (Carbonell and Parteleno-Barehmi, 1999; Gow et al., 2011), increasing empathy and self-awareness (Dogan, 2018), as well as treating PTSD and trauma (Baumgartner, 1986; Bannister, 1990, 1991, 1997; Clarke, 1993; Paivio and Greenberg, 1995; Burge, 1996; Hudgins and Drucker, 1998; Hudgins et al., 2000; Lind et al., 2006). Another study found a significant effect difference between action-based interventions and unstructured support groups for decreasing anxiety, depression, and interpersonal conflict within Latino families (Smokowsky and Bacallao, 2009). Interestingly, while psychodrama does not have much standing in American scientific communities, it has been accredited by the governments and insurance systems of Austria (Ottomeyer et al., 1996), Hungary (Pinter, 2001), and by the European Association of Psychotherapy (Cruz et al., 2018).

Psychodrama is often included as a modality within experiential psychotherapy, along with Gestalt, Existential therapy, Humanistic therapy, and Emotion-Focused Therapy. The efficacy of experiential psychotherapy has been demonstrated through empirical research (Smith et al., 1980; Elliott, 1996, 2001; Elliott and Friere, 2008; Elliott et al., 2013) since the 1980s and suggests it is at least as effective as psychodynamic, cognitive behavioral therapy, and other behavioral therapies (Greenberg et al., 1994, 1998; Greenberg and Paivio, 1998; Greenberg and Malcolm, 2002; Elliott et al., 2013; Greenberg, 2013). Elliott et al. (2004) conducted a meta-analysis of 86 studies which found experiential therapies to be statistically equivalent to talk therapies in their effectiveness. They confidently state that the existing research (in 2004) “is now more than sufficient to warrant a positive valuation of experiential conclusion in four important areas: depression, anxiety disorders, trauma, and marital problems” (p. 423). Greenberg (2013) anchors psychodrama and the Therapeutic Spiral Model of psychodrama within the body of experiential psychotherapies research while highlighting that “there is now solid evidence for the efficacy and effectiveness of experiential therapies” (2013, p. 144).

## Materials and Methods

### Treatment Program and Patients

This study explored the effectiveness of psychodrama group psychotherapy in the treatment of PTSD as implemented in an inpatient addictions treatment center trauma tract at Mirmont Treatment Center in Lima, Pennsylvania. Mirmont is a 115-bed inpatient drug and alcohol facility that primarily treats substance use disorders and co-occurring disorders. The inpatient program includes psychoeducation lectures, yoga, mindfulness groups, psychiatric evaluations, medication management, process groups, individual therapy, case management 12-step support groups, and the following specialty groups: pain management, young adults, relapse prevention, emergency responders, and trauma. Mirmont clients access treatment through state insurance, commercial insurance, their Employee Assistance Program (EAP) or self-pay; though the majority of clients are commercially insured. Mirmont’s trauma tract is a special program for clients who meet all or most of the following criteria: (1) an identifiable trauma history, (2) an expected length of stay of at least two weeks, (3) acceptance of primary diagnosis of substance use disorder, (4) willingness to participate in the trauma program, (5) medical and psychiatric stability, as well as adequate ego strength, containment skills, and internal resourcing, and (6) capacity for ongoing trauma therapy upon discharge.

Patients are assessed by their primary counselor upon admission to the inpatient unit for appropriateness of a referral to the trauma group and/or individual trauma therapy sessions of Eye-Movement Reprocessing and Desensitization (EMDR) therapy which includes the completion of the PTSD Checklist Civilian Version (PCL). The trauma program is composed of a group meeting two times each week for a total of 4.5 h with a maximum of 17 participants in the group. The first group of the week lasts 1.75 h and primarily implements educational sessions, sociometric interventions, psychodrama warm-ups, RTR processes, and psychodramatic letter-writing exercises. The second group of the week is 2.75 h in length and each week includes a psychodrama warm-up, sociometric protagonist and topic selection, a full psychodrama, and sharing. Each psychodrama session was facilitated by a board certified psychodramatist or a psychodrama trainee working under supervision towards their certification. Each group was facilitated with curriculum content from RTR and/or implementation of the TSM clinical map to guide the psychodramatic process.

Upon completion of their final trauma group, discharging patients are invited to complete a post-test PCL assessment with a set of patient satisfaction questions and return it to the front lobby receptionist prior to their discharge. There were a total of 570 participants in the trauma program from October 2017 until February 2019 with 134 groups held. Of these, 483 discharged as scheduled and were offered the opportunity to complete the post-test with additional questions on the level of perceived satisfaction – resulting in a response rate of 27.95% (135 participants). Patients who did not discharge as scheduled from the inpatient program did not have the opportunity to complete the post-test or exit survey. Additionally, data from 49 of the clients were unusable due to incomplete responses or inaccurate completion of the PCL pre-test and/or post-test assessment. This convenience sampling method resulted in a sample of 86 participants with 40 males, 44 females, and 2 transgender individuals. The average age of the participants was 41.34 years old (*SD* = 12.53). The mean number of sessions attended by each participant was 4.73 (*SD* = 1.62) with a range of 2 to 8 and a median of 4. Of these participants, 17 (19.77%) individuals received EMDR processing, 54 (62.79%) were given resourcing only and 15 (17.44%) received no EMDR or resourcing. EMDR resourcing refers to the first two phases of the EMDR protocol which include history taking and positive internal resource development installation (such as imagining a safe place).

### Measure

The PTSD Checklist was used before and after treatment exposure to assess treatment effectiveness or change in PTSD-related symptoms. The PTSD Checklist is a 17-item assessment that asks clients to rate how frequently they experience PTSD symptoms based on DSM-IV-TR criteria. Response sets range for each item range from 1 (‘Not at all/never’) to 5 (‘Extremely/daily or almost daily’) with a maximum composite score of 85. Based on previous clinical studies, the PTSD Checklist appears to exhibit strong internal consistency and reliability with Cronbach’s alpha scores that are equal to or greater than.85 (Wilkins et al., 2011). The Cronbach’s alpha was assessed at.922, which indicates a high level of internal consistency and supports the reliability findings of previous studies.

### Patient Experience and Level of Satisfaction With Psychodrama Group Psychotherapy

A set of questions were added to the PCL post-test assessment to measure the patient’s experience and satisfaction levels with the program group psychotherapy. The items were both Likert-type scale items and open-ended questions. The 11-item Likert-type scale used a response range of 1 (Disagree) to 5 (Agree) and focused the perceived quality and effectiveness of the psychodrama programming. For examples, patients were asked their level of agreement with the following statement, “The services have helped me foster a sense of hope for my recovery” and “The psychodrama sessions helped me to move toward resolution of my trauma.” The open-ended items asked them to expand on some of these same themes in greater depth (i.e., specific aspects of programming you liked or disliked most, areas for improvement, safety and tolerability, and willingness to recommend services to others).

### Data Management and Analysis

Quantitative datum was entered and organized using Microsoft Excel. All of the descriptive and inferential statistics were conducted using IBM SPSS 26.0. Qualitative datum (i.e., open-ended responses on level of perceived satisfaction and effectiveness of program) was recorded in a Microsoft Word document and a content analysis was conducted to examine potential patterns in patient responses (as suggested by Huberman and Miles, 1994). The content analysis was conducted by first reviewing patient responses and identifying common terms or phrases. Second, the common terms or phrases were tallied to determine overall frequency of mentions among the patients. For example, for the open-ended question, “Did you find the services to be helpful?,” the terms or phrases such as “yes,” “yes, it was helpful,” “absolutely,” or “extremely helpful” were grouped together in a “Yes” category and tallied to determine level of agreement with this question. This process was repeated for the other open-ended questions on the assessment (see Table 4).

## Results

### Overall Effectiveness of Treatment Program

The effectiveness of psychodrama group psychotherapy program on reducing overall PTSD and PTSD symptom subscales (i.e., re-experiencing and intrusion, avoidance and numbing, and hyper-arousal) mean scores was assessed using a series of paired-samples *t*-tests. Table 1 includes the results of the paired-sample *t*-tests for each outcome variable. Results show a statistically and clinically significant improvement in overall PTSD symptoms and PTSD subscale mean scores. Specifically, the mean scores for the post-test overall PTSD symptom scores (*M* = 41.66; *SD* = 14.92) were statistically lower than the mean pre-test overall PTSD symptom scores (*M* = 55.91; *SD* = 13.39), *t*(85) = 8.816, *p* < 0.001. The mean difference between the pretest and post-test was 14.25 (*SD* = 14.99) with 95% confidence intervals (CI) [11.04, 17.46], which meets the National Center for PTSD’s standards to be designated as a clinically meaningful change (National Center for Posttraumatic Stress Disorder, 2011; as cited in Lang et al., 2012). Similar improvements were also found for each of the subscales including re-experiencing and intrusion, avoidance and numbing, and hyper-arousal (see Table 1). Interestingly, the percentage decrease in reported symptoms associated with PTSD between the pre and post-test measurement ranged from 25% and 26%.

**TABLE 1:** Results for the Paired-samples *t*-tests for PTSD and PTSD symptom subscale mean scores.

	***M***	***SD***	***Mean Difference (% change)***	***t***	**95% *CI***
Overall PTSD Score			14.24 (−25.49%)	8.816*	11.04–17.46
Pre-test	55.91	13.38			
Post-test	41.66	14.92			
Re-experiencing and Intrusion PTSD Subscale Score			4.09 (−24.65%)	7.110*	2.94–5.23
Pre-test	16.59	4.92			
Post-test	12.50	4.97			
Avoidance and Numbing PTSD Subscale Score			6.09 (−26.00%)	7.968*	4.57–7.61
Pre-test	23.42	6.21			
Post-test	17.33	7.14			
Hyper-arousal PTSD Subscale Score			4.07 (−25.60%)	7.742*	3.02–5.12
Pre-test	15.90	4.53			
Post-test	11.83	4.70			

*^∗^*p* < 0.001.*

### Effectiveness of Treatment Program by Gender, EMDR, and Age

To evaluate potential difference in treatment outcomes by gender identity (male, female, and transgender individual) and EMDR (no EMDR, resourcing only, and EMDR processing), a difference score was created by subtracting pre-test scores from the post-test scores and then a series of one-way ANOVAs were conducted. No statistical differences were found among the difference scores for overall PTSD, *F*(2, 83) = 1.192, *p* = 0.309, *η*^2^ = 0.028, or the re-experiencing and intrusion, *F*(2, 83) = 1.337, *p* = 0.268, *η*^2^ = 0.031; avoidance and numbing, *F*(2, 83) = 0.654, *p* = 0.522, *η*^2^ = 0.016; and hyper-arousal, *F*(2, 83) = 0.874, *p* = 0.421, *η*^2^ = 0.021 subscales by gender identity (see Table 2). Additionally, no statistically significant differences were found for EMDR processing groups as shown in Table 2.

**TABLE 2:** Results of the ANOVAs for Treatment Effectiveness by EMDR resource processing.

	***M***	***SD***	***F***	***df***	***p***	***η ^2^***
Pre/Posttest Overall PTSD Mean Difference Score			0.969	2, 83	0.384	0.023
No EMDR (*n* = 15)	15.90	14.13				
Resourcing only (*n* = 54)	12.59	14.19				
EMDR processing (*n* = 17)	18.06	18.03				
Re-experiencing and Intrusion PTSD Subscale			1.193	2, 83	0.308	0.028
No EMDR (*n* = 15)	4.73	5.36				
Resourcing only (*n* = 53)	3.44	4.89				
EMDR processing (*n* = 17)	5.59	6.51				
Avoidance and Numbing PTSD Subscale			0.641	2, 83	0.529	0.015
No EMDR (*n* = 15)	6.23	7.68				
Resourcing only (*n* = 53)	5.53	6.96				
EMDR processing (*n* = 17)	7.77	7.15				
Hyper-arousal PTSD Subscale			0.594	2, 83	0.554	0.014
No EMDR (*n* = 15)	4.93	3.92				
Resourcing only (*n* = 54)	3.62	4.76				
EMDR processing (*n* = 17)	4.71	6.01				

Additionally, there was no statistically significant correlation between age and any of the mean difference PTSD or subscale scores. Differences in PTSD symptoms were not assessed by number of sessions because the number of sessions attended was largely dictated by insurance providers authorizing additional days of treatment based on specific medical criteria. This often results in clients that have diagnosed with persistent symptoms remaining in treatment longer and clients with decreases in symptoms transitioning to lower levels of treatment. Finally, it is notable that 8 clients (9.3%) demonstrated a reliable (5-10) increase in PTSD symptoms from pretest to posttest and only one (1.16%) client demonstrated a clinically meaningful increase in PTSD symptoms (11 point increase).

### Effectiveness of Treatment Program by EMDR Controlling for Number of Psychodrama Group Psychotherapy Sessions Attended

A set of ANCOVAs was performed to adjust for potential differences in treatment effectiveness by number of psychodrama group sessions attended. The results of the analyses suggest no statistical difference between treatment effectiveness by EMDR, controlling for the number of sessions for overall PTSD, *F*(2, 82) = 0.938, *p* = 0.396, MSE = 227.52, partial *η*^2^ = 0.028; re-experiencing and intrusion subscale, *F*(2, 82) = 1.232, *p* = 0.297, MSE = 28.57, partial*η*^2^ = 0.029; avoidance and numbing, *F*(2, 82) = 0.531, *p* = 0.522, MSE = 51.01*η*^2^ = 0.013; or hyper-arousal, *F*(2, 82) = 0.582, *p* = 0.561, *η*^2^ = 0.014.

### Patient Experience and Level of Satisfaction With Psychodrama Group Psychotherapy

Table 3 lists the descriptive statistics for 85 individuals that completed the exit survey; one participant’s survey was not included because of missing responses. Of note, all of the item means are greater 4.50, which indicates high levels of agreement and positive regard toward each component of the psychodrama group psychotherapy. The results of the content analysis of the open-ended questions suggested that their experiences with the psychodrama group therapy were positive overall with all responding patients (note: 3–5 patients did not provide a response to some of the items) stating that the trauma services they received were helpful, safe, and that they would recommend to others (see Table 4). Patients were also asked which aspect of the trauma services they found most helpful. A large majority explicitly indicated that they found psychodrama to be the most helpful (*n* = 62 or 72.94%) while some participants wrote that “all of the services were helpful” and some highlighted other elements such as EMDR, sociometry warm-ups, sharing their experience, or identifying with others.

**TABLE 3:** Descriptive Statistics for Patient’s Experience and Level of Satisfaction Likert-type scale items.

**Patient Satisfaction Assessment Items (*n* = 85)**	***M***	***SD***
I experienced the facilitator(s) as competent.	4.89	0.58
The services provided a space for me to connect with others about my experiences.	4.85	0.63
I would recommend these services to future clients.	4.83	0.65
I felt emotionally safe during the services.	4.80	0.65
The trauma services were helpful and beneficial to me.	4.80	0.40
The services have helped me foster a sense of hope for my recovery.	4.79	0.47
I increased my knowledge about trauma and my knowledge about myself.	4.74	0.52
The psychodrama sessions helped me identify my own strengths.	4.73	0.59
The psychodrama sessions helped me to express difficult feelings.	4.69	0.66
The psychodrama sessions helped me to move toward resolution of my trauma.	4.64	0.65
The services helped me to manage or reduce my trauma-related symptoms.	4.59	0.76

**TABLE 4:** Frequency Data for Patient’s Experience and Level of Satisfaction Open-ended Questions.

**Open-ended Patient Satisfaction Assessment Questions (*n* = 85)**	**Agreed (%)**	**Disagreed (%)**	**Nonresponse (%)**
Did you find the services to be helpful?	81 (95.29)	0 (0)	4 (4.71)
Did you feel safe in the trauma services?	82 (96.47)	0 (0)	3 (3.53)
Would you recommend the trauma services to others?	80 (94.12)	0 (0)	5 (5.88)

## Discussion

### Interpretations and Implications

#### Quantitative Findings

The findings of this study suggest that trauma-focused psychodrama may be an effective treatment for post-traumatic stress disorder in inpatient substance use treatment centers. Specifically, on average, after 2–3 weeks of participation in the trauma-focused psychodrama tract, PTSD symptoms declined by over 25%. These results support the findings of previous research on psychodrama therapies. Psychodrama and other creative arts approaches may be ideally suited to treat symptoms associated with PTSD because of the emphasis on non-verbal movements and expressions. For example, a 2018 systematic review on PTSD and creative arts therapies (including psychodrama) suggested that decreased symptoms may be related to non-verbal expression or symbolic expression of painful memories or experiences that would have been otherwise difficult to express with words (Baker et al., 2018). Additionally, previous neuroscience studies in the localization of brain function have found that PTSD symptoms are more closely related to brain functions in the right hemisphere and limbic system of trauma survivors (Rauch et al., 1996; van der Kolk, 2014), which is also where creative brain functioning occurs. Further, Malchiodi (2014) proposes that the right brain dominant nature of creative arts therapies position them to be effective for treating PTSD.

Furthermore, van de Kamp et al. (2019) conducted a systematic review and meta-analysis of body- and movement-oriented interventions (BMOI) for PTSD and found a statistically significant decrease in PTSD symptoms. It was also found that cognitive, verbal, and other *top-down* approaches to therapy interface primarily with the prefrontal cortex, while BMOIs engage evolutionarily older brain systems which are impacted by trauma but only marginally accessed through cognition or talking (Ogden et al., 2006; Levine, 2010; van der Kolk, 2014). The *bottom-up* nature of (BMOI), including psychodrama, position them to address the somatic imprints of trauma and include the body, and thus the nervous system, in the therapeutic intervention (Levine, 2010; van der Kolk, 2014).

Finally, psychodrama, the creative arts therapies, and other BMOIs engage the whole person and the whole brain in the therapeutic process. Baker et al. (2018) also suggest that the creative arts therapies are effective for treating PTSD because they renegotiate trauma while providing a sense of containment, control, empowerment, and pleasure through the process of engaging with art. Indeed, the trauma-focused psychodramatic process seems to offer these experiences to participants allowing for right brain activation and expression with containment, control, empowerment, and pleasure. The various qualitative feedback from participants in this study appears to confirm this. For example, one patient stated, “I came to realize during this group that I am worthy of happiness and my past does not define my future.” Another patient stated, “at no time did I feel out of control.” And a third participant commented that, “The environment allowed me to open up fully and safely.”

In terms of the different symptom clusters of PTSD, our analysis found similar decreases in each – Re-experiencing and Intrusions (−24.65%), Avoidance and Numbing (−26.00%), and Hyperarousal (−25.60%). The TSM trauma-focused psychodrama model specifically focuses on safety, containment, and strengths – each of which may be uniquely related to the three PTSD symptom clusters. Theoretically and clinically, containment provides a mechanism for preventing re-enactment and intrusions, safety offers a solution to hyperarousal, and strength-based nature of TSM orients itself on the strengths needed to acknowledge and heal from trauma. The descriptive statistics from participant responses related to feeling safe (4.8/5) and being able to identify their own strengths (4.73/5) may shed light upon the mechanism from which hyperarousal and avoidance and numbing symptoms were alleviated. The open-ended responses also support these conclusions. The experiential nature of psychodrama offers the potential of re-enacting scenes (interpersonal or intrapsychic) related to trauma while create new endings – this effectively provides an avenue for renegotiating the intrapsychic repetitions and re-enactments related to trauma while offering a corrective emotional experience (Giacomucci and Stone, 2018).

#### Qualitative Findings

The qualitative responses and descriptive statistics from the sample highlight that the majority of participants felt emotionally safe and connected to others, while agreeing that the services were beneficial, increased hope, facilitated the expression of feelings, and both helped with identifying strengths and managing trauma-related symptoms (all of which had mean scores above 4.5/5.0; see Table 3). These client responses offer insight into the experience of the process overall and as it relates to the reduction in PTSD symptomology.

The prevalence of positive satisfaction in the surveys of this study seem to reflect outcomes in Dougherty’s (2002) qualitative survey of patient satisfaction of an inpatient program for trauma and dissociation during which participants rated (classical) psychodrama as one of the most useful/helpful of the 21 different treatment interventions of their program. The only interventions that were rated higher were individual sessions with the psychiatrist, individual sessions with the psychotherapist, and trauma group. Dougherty notes that “responses from participants about psychodrama ranged from ‘it scares me’ to ‘it was the turning point for me”’ (2002, p. 103). One might argue that patient satisfaction rates may have been higher had a trauma-focused psychodrama model been utilized with an emphasis on safety and strengths.

#### Safety and Tolerability

The theme of safety is one of the most important considerations in the provision of trauma treatment due to the very nature of trauma and how it can threaten an individual’s wellbeing. In order to avoid retraumatization and to serve as a practical treatment, trauma-focused therapies and psychodrama should be tolerable for clients. In other words, the client must remain within their window of tolerance for the treatment to be useful (Siegel, 2012). The analysis of open-ended survey results revealed that the very idea of seeking help and attending a trauma-focused group was intimidating to many clients but that the trauma-focused group appeared to be a tolerable and an experience that lead to positive affect. For example, the following two client responses demonstrate these point well, “I was so nervous before my first session but instantly felt calm, safe, and at ease in the group” and “I always thought trauma groups had to be painful – I found that not to be truthful.” Another participant commented that psychodrama group psychotherapy “is a wonderful experience that helps you work through trauma in a safe way.”

In some professional circles, psychodrama seems to have developed a reputation to some as retraumatizing. However, based on the responses of the clients, when facilitated from a strengths-based perspective with an orientation to safety, it can be a transformative experience – “it felt safe to express yourself in any manner which caused a peace within.” While many have framed psychodrama as an exposure therapy, none of the psychodrama sessions in this study involved dramatizing a trauma scene. Instead, the psychodramas were focused on strength-based roles, defense mechanisms, and roles of transformation. Psychodrama as a treatment for trauma and PTSD is unique in that the client does not need to tell all the details of their traumatic experiences in order to heal from it. One participant commented on this theme noting – “I was able to release and let go of my trauma without reliving it.”

#### Cohesion, Connection, and Normalizing

Other primary themes that emerged from the open-ended survey items were experiences of group cohesion, connection, and the normalization of their traumatic experiences in a group setting. Some of the participant comments reflect this, for example, “we had a connection and bond with everyone that was very strong.” and “it’s nice knowing that you are not the only one – it allowed me to trust the process.” The nature of group therapy lends itself to layers of mutual aid and connection that would be otherwise unavailable in individual work (Giacomucci, 2020b). The method of facilitation used in the trauma services emphasized connection as a core aspect of group safety. Experiential sociometry exercises were also used prior to psychodrama with the goal of uncovering similarities and shared experiences between group members while establishing group cohesion.

Psychodrama’s group-as-a-whole approach appears to have helped validate each group members’ individual experience. Even if one was not the protagonist of the psychodrama, they reported experiencing therapeutic benefits – *“*in the psychodrama, not only can someone work out and reprocess their past, but I could work out parts of my own past as well.” Another participant’s response suggested “it is helpful and it allows putting your trauma issues out through each other. Every group is healing.” The group size in this study generally ranged between 8–18 participants, but these comments suggest the potential of using psychodrama as a treatment approach with larger audiences. Moreno and Aristotle described this phenomenon as *audience catharsis* or *actor catharsis* – “the greater a spectator’s social and psychodramatic roles correspond to the symbolic roles portrayed on the stage, the greater is the catharsis produced by the drama” (Moreno, 1940, p. 226). Another participant noted that “Even though I was never the protagonist, I feel as though I got as much out of the group as anyone who entered that role.” Some theorize that *mirror neurons* are the neurobiological underpinnings of audience catharsis (Hug, 2007). Psychodrama may be unique in this effect on observers due to its action-based nature.

#### Action vs. Talk

One of the most fundamental differences between psychodrama and other treatment approaches is the involvement of action through experiential methods rather than only talking. While most of the inpatient treatment program is based on traditional talk therapy approaches, the trauma services were entirely experiential. Participants commented on the action-based nature of the group indicating – “I liked that it was also a fun experience,” “Psychodrama was cool!,” and “it used tools to access different parts of our brain (experiential/art exercises) that allowed us to experience trauma safely and differently than we are used to.” These comments from participants demonstrate that not only did they find the psychodrama tolerable and safe, but that some even experienced it as “cool” and “fun.” The experiential nature of psychodrama allows clients to bring their full self, including their body, into the process. Instead of talking about healing from trauma, the healing was put into action in the here-and-now. When asked “which aspect of the trauma services did you find most helpful?,” an overwhelming majority indicated the psychodramas – “the most helpful part was the psychodrama and the interactive exercises.” Another participant noted that “hands down the psychodramas were by far the most amazing part of my recovery thus far, it was actually life changing. I am forever grateful.”

Furthermore, in response to the open-ended survey question, “how could the services be improved?”, 65.88% (*n* = 56) of participants suggested that more psychodrama groups be included in the program. This further depicts that in addition to its effectiveness in treating PTSD, participants overall appreciated the psychodrama process and wanted to participate in more psychodrama. One participant simply responded, “More psychodrama sessions!.” Another stated, “This service is amazing and needs no improvement, only more sessions would be nice.” A third patient stated, “I wish we had the group more often so I had a chance to be the protagonist in psychodrama.”

### Trauma-Focused Psychodrama and PTSD

The present study may also contribute to the discussion on the effectiveness of trauma-focused psychodrama models as compared to classical psychodrama. Ragsdale et al., 1996 study on classical psychodrama as a treatment for veterans with PTSD in an inpatient setting found no change in PTSD symptoms while this inpatient study demonstrated a 25% decrease in PTSD symptomology. While the impetus for the development of TSM and RTR as trauma-focused psychodrama models was based in clinical practice experience, this study may be the first to offer empirical evidence to support their effectiveness compared to classical psychodrama.

In terms of the minority of participants that experienced an increase in PTSD symptoms, there are many factors that could potentially explain this. These factors include an increased awareness of symptoms at the end of their treatment, willingness to disclose symptoms, access to emotions or pain after years of using alcohol or drugs to numb emotions; and reduced defense mechanisms and medications related to their detoxification process. For example, one client noted in their satisfaction survey that “I was not really aware of how much my trauma affected me before the group.”

The results of this study are promising, but some issues still need to be addressed to determine its applicability in other contexts. First, much replication is needed to determine if these treatment effects can be realized in other sub-populations (i.e., outpatient facilities, military and non-military trauma applications). Almost all inpatient PTSD studies that have been published in peer-reviewed journals have focused on veterans which makes it difficult to compare our results due to the findings that veterans with PTSD benefit less from psychotherapy than non-military populations (Watts et al., 2013). More research is needed to explore the impacts of various trauma treatment approaches for inpatient substance use disorder populations.

Second, it is important to note that the models employed in this program included a synthesis of two trauma-focused psychodrama models, the Therapeutic Spiral Model and the Relational Trauma Repair Model, which also need further validation through replication. Future research is needed to explore the effectiveness of each of these models when used independently.

Third, comparative studies are needed to determine if psychodrama group therapy could be used as an alternative or supplemental treatment to more traditional trauma-informed treatment approaches. The closest comparison that we could find to the current programming was a 4-week trauma-focused CBT residential program (Berle et al., 2018), which showed a mean decrease in PTSD (using PCL) at 5.85 (9.32% decrease). In comparison, the current trauma-focused psychodrama program demonstrated a decrease in PTSD of 14.57 points (25.49% decrease). In other words, the current study demonstrated a 2.74 times greater treatment effect on PTSD symptoms than the 4-week trauma-focused residential CBT program researched by Berle et al. (2018). This is, of course, not a perfect comparison as variables were inconsistent between the two studies. While our PTSD checklist pretest mean was 55.8, theirs was 62.78. The CBT program also had a longer length of treatment and was not specific to substance use disorder. Nevertheless, this comparison provides some contextualization of our results compared to a similar study. This comparison is, however, consistent with a large meta-analysis by Elliott et al. (2013) that found the Humanistic-Experiential Psychotherapies (psychodrama, gestalt, emotion-focused, existential, focus-oriented, and body-oriented therapies) to be superior to CBT in their efficacy treating interpersonal difficulties and unresolved relationship issues, including childhood trauma. The study at hand responds to their conclusion that while HEPs appear to be efficacious in the treatment of interpersonal issues, further research is urgently needed to assess HEPs effectiveness in treating PTSD specifically (Elliott et al., 2013).

### Limitations

There are inherently some difficulties in developing research studies focused on psychodrama (Kellermann, 1992; Kipper and Ritchie, 2003; Ridge, 2010). Some psychodrama practitioners suggested that the nature of psychodrama, with “spontaneity-creativity theory” at its heart, makes it impossible to manualize and measure. No two psychodrama groups are ever identical. Furthermore, a variety of clinical interventions are embedded within a single psychodrama group including role playing, role reversal, doubling (at least 4 types of doubling in the literature), and mirroring. The interventions implemented are determined by the clinical judgment of the facilitator and vary from group to group.

There are also many intrinsic difficulties to conducting research in inpatient settings. The nature of inpatient or residential treatment impacts the plausibility of conducting randomized controlled studies due to nonrandom selection and other patient factors (Ragsdale et al., 1996; Johnson and Lubin, 1997; Zappert and Westrup, 2008).

This specific study carries with it multiple additional limitations. First, the use of the PTSD Checklist correlating to DSM-IV diagnostic criteria impacts the generalizability of the outcomes as most are using an updated PTSD checklist. Second, the lack of a true control group for the study makes it difficult to determine if PTSD symptom changes are a result of the trauma program alone, and how much influence other aspects of the inpatient program had on PTSD levels including time-specific outcomes, cohort effects, and cross-treatment contamination. The ability to isolate effects of trauma-focused psychodrama in this study is compromised due to the absence of a control group and participants’ involvement in other inpatient treatment services. Third, the nature of unplanned discharges ruled out the possibility of including these clients in our sample. These individuals included those clients who left treatment against medical advice, AWOL, or due to urgent medical, psychiatric, or behavioral issues did not have the chance to complete post-tests or surveys. Fourth, the lack of follow-up assessment also impacts the validity of our findings and our ability to assess the long-term treatment effects following the treatment exposure. Finally, other extraneous variables impacting the generalizability of our findings include variability of other co-occurring mental health diagnoses, the low response rate, variations in the number of group sessions attended, and levels of participation from clients within each group. In the psychodrama sessions, group members were given the option to volunteer as the psychodrama protagonist, play an auxiliary role in the psychodrama, or observe.

### Future Research

There is a great need for more rigorous research on psychodrama as a psychotherapy approach with PTSD and other mental health disorders (Orkibi and Feniger-Schaal, 2019). Very few randomized controlled studies exist in the psychodrama research base. One reason for this may include the absence of psychodrama in many places of higher education and training, which would lend itself to more exposure to student population and increase the likelihood it will receive research support and grants.

Additionally, more research is needed in general to determine the treatment efficacy psychodrama psychotherapy approaches with non-veteran inpatient substance abuse populations that have been diagnosed with PTSD. Replication research is needed to further validate psychodrama psychotherapy as alternative or supplemental trauma-informed therapeutic approach in a variety of subpopulations and practical settings.

Future research could also explore the possible differences in efficacy of psychodrama based on the roles held by participants during group therapy sessions. Comparing the change in symptomology between participants who were the protagonist, supportive roles, and observers would illuminate another aspect of psychodrama’s therapeutic effect and the potential distribution of effect based on level of active engagement.

## Conclusion

This study highlights trauma-focused psychodrama as a potentially effective treatment approach for the treatment of PTSD. While more research is needed to explore psychodrama psychotherapy’s effectiveness, it offers a promising alternative to traditional talk therapies for individuals that have experienced trauma. Many clients indicate that other types of trauma therapy are painful or sometimes even intolerable. The findings in this study suggest the opposite, that participants found the psychodrama as tolerable and even “fun.” Psychodrama originated nearly 100 years ago and seems to have been largely neglected until recently by trauma therapists. The findings of this study offer merit to the potential revitalization of psychodrama as an approach to treating trauma.

## Data Availability Statement

The datasets for this manuscript are not publicly available due to patient confidentiality. Requests to access the datasets should be directed to the primary author.

## Ethics Statement

The studies involving human participants were reviewed and approved by Pennsylvania State University Brandywine Campus IRB. The patients/participants provided their written informed consent to participate in this study.

## Author Contributions

SG and JM collaborated on the design of the study and writing of the manuscript. SG facilitated the collection of research data and wrote the first draft of the manuscript. JM conducted the statistical analyses, wrote most of the results section, and created the tables.

## Conflict of Interest

SG is contracted to provide clinical services by Mirmont Treatment Center, a company that may be affected by this research. To mitigate potential conflicts of interest, SG did not conduct the data analyses for this study.

The remaining author declares that the research was conducted in the absence of any commercial or financial relationships that could be construed as a potential conflict of interest.
